# Impact of changes in physical activity and incident fracture after acute ischemic stroke

**DOI:** 10.1038/s41598-023-44031-8

**Published:** 2023-10-04

**Authors:** Dae young Cheon, Kyung-Do Han, Jeen Hwa Lee, Kyung-Ho Yu, Bo Young Choi, Minwoo Lee

**Affiliations:** 1 Division of Cardiology, Department of Internal Medicine, Dongtan Sacred Heart Hospital, Hwaseong, Korea; 2 Department of Statistics and Actuarial Science, Soongsil University, Seoul, Korea; 3 Department of Neurology, Hallym University Sacred Heart Hospital, Anyang, Korea; 4 Department of Physical Education, Hallym University, Chuncheon, Korea

**Keywords:** Neurology, Neurological disorders, Cerebrovascular disorders, Stroke

## Abstract

Stroke survivors are at an increased risk of falls and fractures. Physical activity is inversely associated with the fracture risk in the general population. However, the association between incident fracture risk and changes in habitual physical activity before and after an index stroke remains unclear. This study attempted to explore the association between incident fracture risk and changes in physical activity after stroke. Using the claims database of the National Health Insurance Program in Korea, participants with their first ischemic stroke between 2010 and 2016 were enrolled in the study. The participants were divided into four groups according to changes in physical activity habits evaluated using two consecutive self-reported questionnaires before and after stroke, if available: persistent non-exercisers, exercise dropouts, new exercisers, and persistent exercisers. The primary outcome was a composite of vertebral, hip, and other fractures. We performed multivariable Cox proportional hazard regression analysis and provided adjusted hazard ratios and 95% confidence intervals for each outcome. Among 202,234 participants included from 1,005,879 datasets, 16,621 (8.22%) experienced any type of fracture as the primary outcome. After multivariable adjustment, exercise dropouts (n = 37,106), new exercisers (n = 36,821), and persistent exercisers (n = 74,647) had a significantly reduced risk of any type of fracture (aHR 0.932, 95% CI 0.893–0.973; aHR 0.938, 95% CI 0.900–0.978; aHR 0.815, 95% CI 0.780–0.852, respectively) compared to the persistent non-exercisers (n = 53,660). Furthermore, regardless of pre-stroke exercise status, those who exercised ≥ 1000 metabolic equivalents of tasks post-stroke had a significantly reduced risk of fractures. Initiating or continuing moderate-to-vigorous regular physical activity after acute ischemic stroke was associated with a significantly lower risk of incident fractures, including hip, vertebral, and other types.

## Introduction

Stroke is a leading cause of disability, and stroke survivors are at an increased risk of fractures due to reduced bone mineral density and an elevated risk of falls. Previous studies have suggested that stroke increases the risk of fracture by up to 4 times compared with the general population^1–3^. More than two-thirds of stroke survivors with mild-to-moderate disability experience falls during the first 6 months after acute stroke^4^. Post-stroke fractures not only increase the degree of disability and mortality, but also increase the burden on the family and society.

Physical activity is one of the most important modifiable factors associated with fracture risk, along with other lifestyle behaviors^5^. Previous studies have shown that initiating or maintaining regular exercise may prevent fractures in older patients^6,7^. Furthermore, regular exercise after stroke is an important modality that reduces multiple risk factors for post-stroke fractures. Thus, regular exercise is recommended for stroke survivors to maintain the bone mineral density and manage falls^8,9^. However, it is still not well known whether initiating or continuing exercise after stroke can prevent fracture risk via the aforementioned mechanisms.

In the present study, we investigated the association between changes in physical activity after acute ischemic stroke and incident fracture risk using the Korean National Health Insurance Services (K-NHIS) database.

## Material and methods

### Data source and study population

We utilized a nationwide medical dataset from the Korean National Health Insurance Service (K-NHIS), a single insurer administered in Korea that covers more than 97% of the Korean population. The K-NHIS provides a biennial general health examination to all individuals aged 40 years and older, which includes demographic, clinical, and laboratory workups and a standard questionnaire regarding lifestyle behaviors, such as exercise and drinking and smoking habits^10^. Furthermore, the K-NHIS retains medical claims data, including inpatient and outpatient usage of medical services, prescription records, and diagnostic codes according to International Classification of Diseases (ICD)-10. The study was approved by the Institutional Review Board (IRB) of the Dongtan Sacred Heart Hospital (IRB number: HDT 2023-01-010). The participants who underwent national health checkups provided written informed consent for the use of their data for research purposes. This study was designed and conducted according to the Strengthening the Reporting of Observational Studies in Epidemiology Reporting Guidelines.

**Figure 1:**
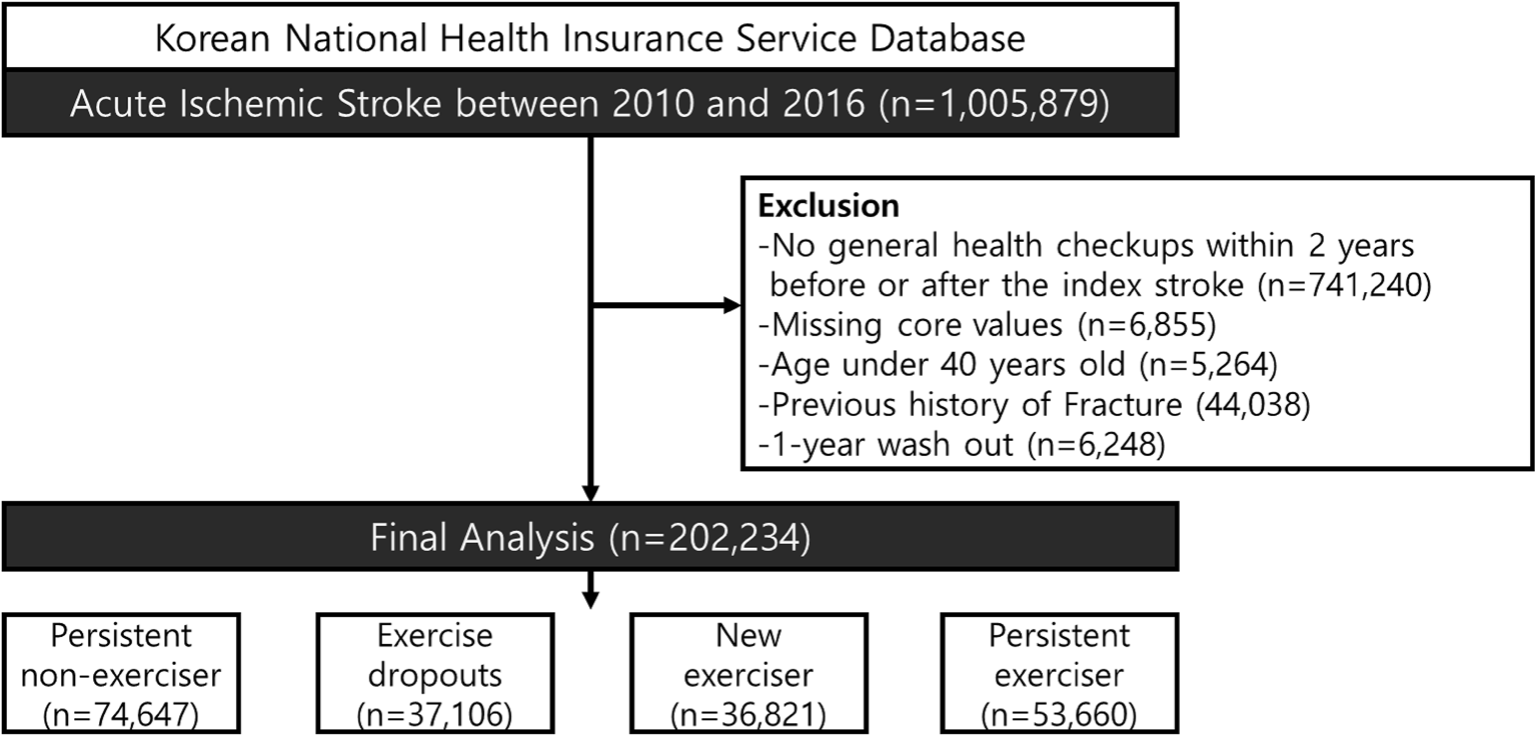
Flowchart of study.

From the K-NHIS database of the entire Korean population, we collected data from participants who were newly diagnosed with acute ischemic stroke between January 2010 and December 2016. Acute ischemic stroke was defined using the 10th edition diagnostic codes of the ICD-10 I63 and I64 upon admission, combined with brain imaging using either computed tomography or magnetic resonance imaging. This definition was validated with high accuracy in previous studies^11–14^. Among the 1,005,879 participants, those who did not undergo a general health examination with a standard questionnaire 2 years before (541,463 participants) and after (199,777 participants) the index stroke were excluded. Among the remaining 264,639 patients, those with missing questionnaires (6855 participants), those younger than 40 years (5264 participants), those with previous diagnoses of any fracture (44,038 participants), and those who experienced any fracture within 1 year after their second examination (6248 participants) were excluded to reduce potential reverse causality. Ultimately, 202,234 participants were included in the analysis. (Fig. 1).

### Definition of exercise habit changes

Information on exercise habits was obtained from two consecutive standardized self-report questionnaires on the intensity and frequency of physical activity over the past 12 months. This questionnaire was based on the International Physical Activity Questionnaire, developed by the World Health Organization and validated for the Korean population^15^. The physical activity section of the questionnaire included three questions regarding the frequency of light, moderate, and vigorous exercise per week. Light-intensity exercise was defined as walking or sweeping carpets for ≥ 30 min. Moderate intensity included bicycle riding, walking at a brisk pace, or playing sports, such as tennis, for ≥ 30 min. Vigorous-intensity exercise was defined as running, climbing, quick bicycle riding, or aerobic workouts for ≥ 20 min. Regular physical activity was defined as moderate or vigorous exercise performed at least once a week. The participants were then assigned to one of the following four groups according to changes in exercise habits before and after the index stroke: (1) persistent non-exercisers, (2) exercise dropouts, (3) new exercisers, and (4) persistent exercisers. To evaluate the influence of energy expenditure on outcomes, we stratified regular physical activity groups according to the degree of energy expenditure using metabolic equivalents of tasks (METs). The multipliers for light, moderate and vigorous-intensity exercise were 2.9, 4.0, and 7.0, respectively for the calculation of energy expenditure. Total energy expenditure, defined as the summation of METs multiplied by the frequency of each intensity exercise with the minimum duration, was divided into < 1000 and ≥1000 Met-min/week for subgroup analysis^16^.

### Covariates and outcome variables

Information on all covariates was collected based on the second health examination. We obtained demographic data, including age, sex, years of education, height, weight, and waist circumference. Lifestyle risk behaviors, including smoking and drinking habits, were also recorded. Smoking status was divided into never smokers, ex-smokers, and current smokers based on their responses to the questionnaire. Alcohol consumption status was divided into alcohol users (alcohol consumption) and non-alcohol users. Comorbidities were operationally defined based on medical claims according to the ICD-10 codes (hypertension, I10–I13 or I15; diabetes mellitus, E11–E14; dyslipidemia, E78^17^; chronic kidney diseases: glomerular filtration rate, calculated by the Modification of Diet in Renal Disease, lower than 60 ml/min/1.73 m^2^). Those who were medical benefit beneficiaries and were included in the lowest income level quartile were defined as low-income level. Furthermore, systolic/diastolic blood pressure and laboratory results, including random glucose, glomerular filtration rate, and total cholesterol, were obtained during ambulatory health examination visits after the index stroke.

The primary composite outcome of this study was the incidence of any fracture after ischemic stroke, including the vertebral, hip, and other types of fractures. Vertebral fracture was defined when both ICD codes (S12.0, S12.1, S12.2, S22.0, S22.1, S32.0, M48.4, or M48.5) and at least 2-time outpatient visits were confirmed^18–20^. Hip fracture was defined when both ICD codes (S72.0, S72.1, or 72.2) and at least one hospital admission were confirmed^19,21–23^. Other fractures were defined when ICD codes (S42.0, S42.2, S42.3, S52.5, S52.6, S82.3, S82.5, and S82.6) and at least 2-time outpatient visits were confirmed^20^. The secondary outcomes included the occurrence of vertebral and hip fractures. The date of the second health examination was defined as the index date, and the participants were followed up until the occurrence of the primary outcome or December 31, 2019, whichever came first.

### Statistical analysis

We used a complete case analysis approach. Participants with missing or incomplete data were excluded from the study. The baseline and demographic characteristics were presented using the mean ± standard deviation for continuous variables and numbers with frequencies for categorical variables. Differences between study groups were compared using one-way analysis of variance or the Chi-square test, as appropriate. The annual incidence of the outcome variables was calculated by dividing the number of outcome events by 1,000 person-years. Multivariable Cox proportional hazard regression analyses were conducted to estimate the crude and adjusted hazard ratios (HR) and 95% confidence intervals (CI) for the association between changes in exercise habits and the incidence of fractures. The covariates adjusted for the analysis were determined according to the priori knowledge with sequential steps as follows: Model 1 for age and sex and Model 2 for age, sex, comorbidities (hypertension, dyslipidemia, diabetes mellitus, and chronic kidney diseases), and smoking, alcohol consumption, and low-income status. The persistent non-exercisers group were used as the reference group. Subgroup analyses were performed according to age (40–65, 65 + years), sex, and the presence of other comorbidities. To identify whether the degree of exercise was associated with incident fractures, we stratified the post-stroke exercise groups into two subgroups by MET-min/week with a cutoff of 1000.

Statistical analyses were performed using SAS 9.4 software (SAS Institute, Cary, NC, USA), and 2-sided *p* < 0.05 were considered statistically significant.

### Ethical approval

The study was approved by the Institutional Review Board (IRB) of the Dongtan Sacred Heart Hospital (IRB number: HDT 2023-01-010).

## Results

A total of 202,234 patients (mean age, 64.52 ± 10.42; male, 54.48%) were included in our analyses. The number and percentages of persistent non-exercisers, exercise dropouts, new exercisers, and persistent exercisers were 74,467 (36.9%), 37,106 (18.3%), 36,821 (18.2%), and 53,660 (26.5%), respectively. The baseline and demographic characteristics of each group are presented in Table 1. Compared with persistent non-exercisers, persistent exercisers tended to be younger, more likely to be male, alcohol users, and less likely to have diabetes, hypertension, or chronic kidney disease. There were no clinically significant differences in laboratory results among the groups.

**Table 1:** Baseline characteristics and incident fractures according to the changes in exercise habit in patients with ischemic stroke.

	Total (n = 202,234)	Persistent non-exerciser (n = 74,647)	Exercise dropouts (n = 37,106)	New exerciser (n = 36,821)	Persistent exerciser (n = 53,660)	*P*-value
Baseline characteristics						
Age at stroke onset	63.59 ± 10.42	65.86 ± 10.28	64.29 ± 10.2	62.82 ± 10.24	60.45 ± 10.01	< 0.001
Sex, male	110,171 (54.48)	34,950 (46.82)	20,443 (55.09)	19,905 (54.06)	34,873 (64.99)	< 0.001
Current smoker	25,434 (12.58)	9402 (12.6)	4492 (12.11)	4737 (12.86)	6803 (12.68)	0.013
Any alcohol consumption	52,796 (26.11)	14,261 (19.1)	8584 (23.13)	10,082 (27.38)	19,869 (37.03)	< 0.001
Low income	33,434 (16.96)	13,186 (18.13)	6137 (16.98)	6263 (17.42)	7848 (15.01)	< 0.001
Obesity	78,947 (39.04)	29,124 (39.02)	14,386 (38.77)	14,478 (39.32)	20,959 (39.06)	0.498
Body mass index	24.28 ± 3.11	24.22 ± 3.27	24.24 ± 3.11	24.32 ± 3.08	24.34 ± 2.9	< 0.001
Diabetes mellitus	51,307 (25.37)	20,067 (26.88)	9761 (26.31)	9158 (24.87)	12,321 (22.96)	< 0.001
Hypertension	130,097 (64.33)	50,290 (67.37)	24,213 (65.25)	23,371 (63.47)	32,223 (60.05)	< 0.001
Dyslipidemia	112,013 (55.39)	41,121 (55.09)	20,684 (55.74)	20,578 (55.89)	29,630 (55.22)	0.030
Chronic kidney disease	25,012 (12.37)	10,943 (14.66)	4785 (12.9)	4243 (11.52)	5041 (9.39)	< 0.001
Random glucose	105.64 ± 29.06	106.36 ± 31.04	106.29 ± 29.67	105.07 ± 28.13	104.58 ± 26.23	< 0.001
Total cholesterol	181.17 ± 41.59	182.18 ± 41.84	180.69 ± 41.68	180.87 ± 41.73	180.31 ± 41.03	< 0.001
Glomerular filtration rate	84.19 ± 43.79	82.84 ± 38.78	83.7 ± 42.96	84.86 ± 45.49	85.97 ± 49.33	< 0.001
Systolic blood pressure	126.95 ± 15.26	127.7 ± 15.76	127.13 ± 15.4	126.69 ± 15.08	125.97 ± 14.5	< 0.001
Diastolic blood pressure	77.16 ± 9.85	77.13 ± 10.01	77.13 ± 9.91	77.21 ± 9.77	77.19 ± 9.65	0.522
Incident fracture						
All types of fracture	16,621 (8.22)	7611 (10.2)	3117 (8.4)	2925 (7.94)	2968 (5.53)	< 0.001
Vertebral fracture	8399 (4.15)	4093 (5.48)	1593 (4.29)	1443 (3.92)	1270 (2.37)	< 0.001
Hip fracture	2356 (1.16)	1201 (1.61)	425 (1.15)	390 (1.06)	340 (0.63)	< 0.001

**Figure 2:**
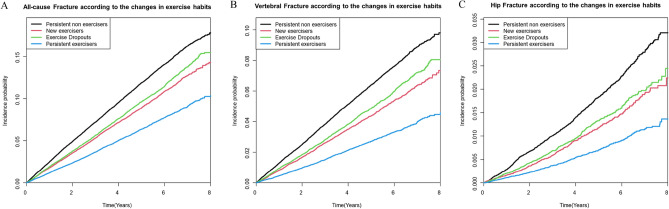
Incidence probability of all-cause fracture (**A**, *P* < 0.001), vertebral fracture (**B**, *P* < 0.001) and hip fracture (**C**, *P* < 0.001) after ischemic stroke according to change of physical activity habits. *Adjusted for age, sex, alcohol consumption, smoking, income, diabetes, hypertension, dyslipidemia and chronic kidney disease.

During a mean follow-up of 4.13 ± 2.03 years, there were 16,621 (8.22%) primary composite outcomes (any types of fractures). Furthermore, 8399 (4.15%) and 2356 (1.16%) participants had vertebral and hip fractures, respectively. Compared with persistent non-exercisers, those who initiated or continued regular exercise after stroke had a significantly lower risk of all types of fractures (new exercisers, aHR 0.932; 95% CI 0.893–0.973, persistent exercisers, aHR 0.815; 95% CI 0.780–0.852, Table 2). Furthermore, those who failed to continue their exercise habits and those who dropped out had a mild but significantly lower risk of all types of fractures than those who did not. (aHR 0.938; 95% CI 0.900–0.978). Consistent findings were observed for both vertebral and hip fractures, except that the new exerciser group did not show a significantly reduced risk of hip fractures. Kaplan–Meier curves demonstrated significant differences in primary and secondary outcomes between the groups according to the status of exercise habits changes. (Fig. 2).

**Table 2:** Risk for All-cause fracture, vertebral fracture and hip fracture according to the changes in exercise habit.

	Number of patients	Number of events	IR (per 1000 PY)	aHR (95% CI) Model 1	aHR (95% CI) Model 2
All-cause fracture					
Persistent non-exerciser	74,647	7611	25.22	1 (Ref)	1 (Ref)
Exercise dropouts	37,106	3117	20.34	0.933 (0.895, 0.973)	0.938 (0.900, 0.978)
New exerciser	36,821	2925	18.88	0.925 (0.887, 0.966)	0.932 (0.893, 0.973)
Persistent exerciser	53,660	2968	13.15	0.800 (0.766, 0.836)	0.815 (0.780, 0.852)
Vertebral fracture					
Persistent non-exerciser	74,647	4093	13.18	1 (Ref)	1 (Ref)
Exercise dropouts	37,106	1593	10.13	0.913 (0.861, 0.967)	0.918 (0.866, 0.973)
New exerciser	36,821	1443	9.08	0.894 (0.841, 0.949)	0.900 (0.847, 0.956)
Persistent exerciser	53,660	1270	5.52	0.703 (0.659, 0.750)	0.716 (0.671, 0.764)
Hip fracture					
Persistent non-exerciser	74,647	1201	3.77	1 (Ref)	1 (Ref)
Exercise dropouts	37,106	425	2.65	0.848 (0.759, 0.948)	0.861 (0.771, 0.963)
New exerciser	36,821	390	2.41	0.881 (0.785, 0.989)	0.904 (0.805, 1.014)
Persistent exerciser	53,660	340	1.46	0.726 (0.641, 0.822)	0.768 (0.678, 0.870)

*IR* incidence rate, *aHR* adjusted hazard ratio, *PY* person-years.

^#^Model 1: age and sex-adjusted, Model 2: Model 1 + smoking status, alcohol status, low income, history of diabetes, hypertension, dyslipidemia and chronic kidney disease adjusted.

Subgroup analyses were performed according to age, sex, and comorbidities. Although consistent findings were observed throughout the age and comorbidity subgroups, a significant interaction was observed between the sex groups. (Table 3 and Supplemental Table S1). The beneficial effects of exercise were not prominent in female groups; only persistent exercisers showed a significantly reduced risk of all types of fractures, and new exercisers or exercise dropouts were not associated with a reduced risk of incident fractures. Additional analyses were performed to determine the effect of the degree of exercise on the risk of fractures. Overall, regardless of pre-stroke exercise habits, exercising ≥ 1000 METs-min/week after acute stroke was associated with a lower risk of fractures compared with no exercise or < 1000 METs-min/week. However, among those who exercised < 1000 METs-min/week, exercising ≥ 1000 METs-min/week post-stroke was not particularly beneficial compared to working < 1000 METs-min/week post-stroke (Supplemental Table S2).

**Table 3:** Subgroup analyses according to the age and sex group and risk for all-cause fracture.

	Exercise habit change status	Number of patients	Number of events of all-cause dementia	IR (per 1000 PY)	aHR (95% CI) Model 1	aHR (95% CI) Model 2
Male	Persistent non-exerciser	34,950	2349	16.90	1 (Ref)	1 (Ref)
	Exercise dropouts	20,443	1118	13.40	0.844 (0.786, 0.906)	0.851 (0.793, 0.914)
	New exerciser	19,905	1053	12.64	0.863 (0.802, 0.928)	0.871 (0.810, 0.937)
	Persistent exerciser	34,873	1386	9.41	0.733 (0.686, 0.784)	0.752 (0.703, 0.804)
Female	Persistent non-exerciser	39,697	5262	32.32	1 (Ref)	1 (Ref)
	Exercise dropouts	16,663	1999	28.63	0.981 (0.932, 1.033)	0.985 (0.936, 1.038)
	New exerciser	16,916	1872	26.12	0.956 (0.907, 1.008)	0.963 (0.913, 1.015)
	Persistent exerciser	18,787	1582	20.18	0.845 (0.798, 0.894)	0.856 (0.809, 0.906)
p for interaction					< 0.001	0.002
40–64 years	Persistent non-exerciser	34,950	1,254	8.87	1 (Ref)	1 (Ref)
	Exercise dropouts	20,443	595	7.02	0.862 (0.782, 0.950)	0.873 (0.792, 0.963)
	New exerciser	19,905	547	6.47	0.881 (0.797, 0.975)	0.892 (0.807, 0.987)
	Persistent exerciser	34,873	600	4.02	0.655 (0.594, 0.722)	0.675 (0.612, 0.745)
≥ 65 years	Persistent non-exerciser	39,697	2,839	16.78	1 (Ref)	1 (Ref)
	Exercise dropouts	16,663	998	13.77	0.940 (0.875, 1.011)	0.943 (0.877, 1.013)
	New exerciser	16,916	896	12.07	0.897 (0.832, 0.967)	0.900 (0.835, 0.971)
	Persistent exerciser	18,787	670	8.29	0.742 (0.681, 0.808)	0.747 (0.686, 0.814)
p for interaction					0.198	0.341

*IR* incidence rate, *aHR* adjusted hazard ratio, *PY* person-years.

^#^Model 1: age and sex adjusted, Model 2: Model 1 + smoking status, alcohol status, low income, history of diabetes, hypertension, dyslipidemia and chronic kidney disease adjusted.

## Discussion

In this nationwide cohort study, we observed the following findings: first, during a median of 4.13 years after stroke, 8.22% of the patients experienced incident fractures. As expected, the incidence of fractures was higher in the female patients. Second, both the initiation and continuation of regular exercise after acute stroke were significantly associated with a reduced risk of all types of fractures. Exercise dropouts after stroke were also mildly but significantly associated with a reduced risk of fracture. Third, in female groups, the impact of post-stroke exercise was less prominent and exercise dropouts or new exercisers were not associated with a reduced fracture risk. Fourth, exercising ≥ 1000 MET-min/week, namely moderate-to-vigorous exercise, was significantly associated with a reduced risk of fractures than mild-intensity physical activity.

In our study, the cumulative incidence of fracture in post-stroke survivors was 8.22% during a median of 4.13 years, which is slightly lower than that in the multicenter stroke registry of Korea, which reported a 13% cumulative incidence at 4 years^24^. While mild-to-moderate stroke patients had the highest cumulative incidence in that study, those who were either independent or infirm enough to be bedridden had a lower incidence of fractures. As our study population mostly consisted of independent to mild stroke patients who were able and willing to visit ambulatory health examinations provided by the government after stroke, our population may have a healthier lifestyle, which may explain the lower incidence of fractures of any kind compared to the multicenter prospective stroke registry study. The incidence of fractures was almost 1.6-fold higher in our stroke cohort than in the general Korean population without a previous history of stroke. Overall, the incidence of fractures in the Korean stroke population was slightly higher than that in the Caucasian population. US and Canadian studies have shown the 2-year incidence of fractures in stroke survivors to be 1.7–6.1% in recent studies^25,26^.

Stroke survivors are at an elevated risk of fractures on their paretic sides, most often in the hip or vertebrae. Specifically, ambulatory stroke survivors, such as our study population, often have poor balance, lower bone mass, and disuse osteoporosis in the paretic arm or legs^27,28^. Therefore, fragile fractures are common in stroke survivors. Furthermore, specific neurological impairments such as hemineglect and attention deficits may increase the risk of falls and subsequent fractures^27,28^. Considering this, proper physical activity and rehabilitation after a stroke may not only improve functional outcomes, but also reduce the chances of falls among the stroke population.

Our results regarding the reduced risk of fracture in physically active stroke survivors are consistent with the findings of previous studies. However, most studies have only assessed the status of physical activity at a single time point, rather than the changes before and after the index stroke. Furthermore, no randomized clinical trials have been conducted with fracture as an outcome because they require a large sample size and follow-up duration, as fracture incidence is not as high^29^. Several studies have been conducted to determine whether exercise could reduce the risk factors for fractures, such as fall frequency, balance, muscle mass, and bone mineral density, which may reflect future fracture risk^30–34^. Furthermore, even for stroke survivors, the benefits of physical activity may outweigh the potential injuries caused by exercise. In our study, moderate-to-vigorous exercise after stroke was associated with a reduced risk of fracture. Furthermore, regular physical activity before stroke onset also had protective effects, even when participants failed to continue regular physical activity after the stroke. These findings are in line with those of previous studies in the general population without any history of stroke, which report that habitual exercise changes are associated with incident fractures^6^.

The identified sex differences in fracture incidence and reduced protective effects of exercise were consistent with those of previous studies. Females have a higher prevalence of osteoporosis^35^ and tend to have more severe neurological deficits and less recovery from stroke^36^. Furthermore, even with regular physical activity, the degree of increase in muscle mass and bone density is limited in females. As muscle mass and bone density play major roles in protecting bones after falls or trauma, physical activity may have less of an impact on female patients^37,38^.

Our study had several limitations. First, as our database was based on the claims dataset of the national insurance system, we lacked important clinical variables associated with ischemic stroke, including stroke severity, antithrombotic use, and discharge status, which may affect the risk of fractures after stroke. Second, as our study only recruited participants who were both willing and able to attend ambulatory health examinations before and after stroke, our study population may be limited to participants with mild stroke with functional independence. Furthermore, most participants in our study were ambulatory post-stroke and capable of completing the questionnaires. This baseline functional capability suggests that they might have engaged in healthier lifestyle behaviors compared to the broader stroke survivor population, introducing a potential for bias. However, given the expansive scale of our study population, any such bias is anticipated to be minimal. Even so, considering the consistent functional status across our cohort, it's imperative to highlight that our findings might not fully extend to all stroke survivors, especially those with greater functional impairments. Third, exercise habits in our study were based on self-reports, which may not provide an exact representation of the actual duration and intensity of the exercise. This method might also not specify whether the exercise was part of a structured rehabilitation program or initiated independently by the participants. Nonetheless, we regard rehabilitation exercises as a subset of physical activity. Given that dedicated participation in rehabilitation exercises is postulated to aid in fracture prevention following a stroke, we chose not to differentiate explicitly between general physical activity and targeted rehabilitation exercises. Despite these inherent limitations, this large-scale nationwide cohort study used real-world data to reveal the effect of changes in exercise habits on the risk of subsequent fractures after stroke.

## Conclusion

Initiating or continuing moderate-to-vigorous regular physical activity after acute ischemic stroke is associated with a significantly lower risk of incident fractures, including hip, vertebral, and other types. The promotion of exercise among ambulatory stroke survivors may reduce the risk of fractures.

### Supplementary Information

Supplementary Tables.

## Data Availability

The data that support the findings of this study are available from National Health Insurance Sharing Service of Korea but restrictions apply to the availability of these data, which were used under license for the current study, and so are not publicly available. Data are however available from the corresponding author Minwoo Lee, upon reasonable request and with permission of National Health Insurance Sharing Service of Korea.
