# Supplementary material for: Impact of changes in physical activity and incident fracture after acute ischemic stroke
Source: Sci Rep. 2023 Oct 4;13:16715. doi: 10.1038/s41598-023-44031-8 (PMC10551008; doi:10.1038/s41598-023-44031-8)
Supplement: Supplementary file 1 — Supplementary Tables. [file 41598_2023_44031_MOESM1_ESM.docx]

| **Supplemental Table 1. Subgroup analyses according to the comorbidities and life style behaviors. and risk for all-cause fracture** | | | | | | |
| --- | --- | --- | --- | --- | --- | --- |
| Subgroup | |  | | | | p for  interaction |
|  |  | Persistent non-exerciser | New exerciser | Exercise dropouts | Persistent exerciser |  |
| Smoking | No | 1(Ref.) | 0.945(0.903,0.988) | 0.946(0.905,0.988) | 0.824(0.787,0.863) | 0.165 |
|  | Yes | 1(Ref.) | 0.814(0.704,0.942) | 0.865(0.749,0.998) | 0.730(0.633,0.841) |  |
| Alcohol Consumption | No | 1(Ref.) | 0.941(0.898,0.986) | 0.946(0.904,0.990) | 0.834(0.794,0.875) | 0.184 |
|  | Yes | 1(Ref.) | 0.876(0.787,0.976) | 0.885(0.791,0.990) | 0.740(0.672,0.816) |  |
| Low income | Other | 1(Ref.) | 0.952(0.907,0.998) | 0.937(0.894,0.982) | 0.816(0.777,0.856) | 0.241 |
|  | Low | 1(Ref.) | 0.856(0.776,0.945) | 0.945(0.860,1.039) | 0.813(0.737,0.897) |  |
| Diabetes Mellitus | No | 1(Ref.) | 0.919(0.874,0.967) | 0.928(0.883,0.975) | 0.809(0.769,0.852) | 0.686 |
|  | Yes | 1(Ref.) | 0.969(0.893,1.050) | 0.965(0.892,1.044) | 0.830(0.763,0.902) |  |
| Hypertension | No | 1(Ref.) | 0.899(0.831,0.973) | 0.931(0.862,1.006) | 0.827(0.767,0.891) | 0.581 |
|  | Yes | 1(Ref.) | 0.947(0.900,0.997) | 0.941(0.896,0.989) | 0.808(0.766,0.852) |  |
| Dyslipidemia | No | 1(Ref.) | 0.948(0.889,1.011) | 0.956(0.899,1.018) | 0.823(0.772,0.879) | 0.828 |
|  | Yes | 1(Ref.) | 0.920(0.869,0.975) | 0.924(0.873,0.977) | 0.808(0.763,0.857) |  |
| Chronic Kidney disease | No | 1(Ref.) | 0.931(0.888,0.975) | 0.941(0.899,0.985) | 0.810(0.772,0.849) | 0.825 |
|  | Yes | 1(Ref.) | 0.942(0.846,1.048) | 0.924(0.835,1.024) | 0.850(0.760,0.950) |  |

| **Supplemental Table 2. Subgroup analyses according to the pre- and post-stroke** MET-min/wk | | | | | | |
| --- | --- | --- | --- | --- | --- | --- |
| Pre MET-min/wk | Post MET-min/wk | Number of patients | Number of events | IR  (per 1,000PY) | aHR*(95% CI) Model 1 | aHR*(95% CI) Model 2 |
| **All-cause Fracture** |  |  |  |  |  |  |
| Non-exerciser | Non-exerciser | 74,647 | 7,611 | 25.22 | 1(Ref.) | 1(Ref.) |
|  | MET-min/wk <1,000 | 20,682 | 1,679 | 19.34 | 0.955(0.905,1.007) | 0.963(0.913,1.016) |
|  | MET-min/wk ≥1,000 | 16,139 | 1,246 | 18.29 | 0.888(0.837,0.943) | 0.894(0.842,0.949) |
| MET-min/wk <1,000 | Non-exerciser | 21,304 | 1,747 | 19.74 | 0.924(0.877,0.973) | 0.929(0.882,0.979) |
|  | MET-min/wk <1,000 | 16,824 | 844 | 11.85 | 0.777(0.723,0.835) | 0.792(0.736,0.851) |
|  | MET-min/wk ≥1,000 | 11,128 | 606 | 12.84 | 0.798(0.734,0.867) | 0.812(0.747,0.883) |
| MET-min/wk ≥1,000 | Non-exerciser | 15,802 | 1,370 | 21.16 | 0.945(0.892,1.001) | 0.950(0.897,1.006) |
|  | MET-min/wk <1,000 | 9,472 | 592 | 14.74 | 0.837(0.770,0.911) | 0.850(0.782,0.925) |
|  | MET-min/wk ≥1,000 | 16,236 | 926 | 13.79 | 0.800(0.747,0.858) | 0.817(0.762,0.875) |
| **Vertebral Fracture** | |  |  |  |  |  |
| Non-exerciser | Non-exerciser | 74,647 | 4,093 | 13.18 | 1(Ref.) | 1(Ref.) |
|  | MET-min/wk <1,000 | 20,682 | 837 | 9.40 | 0.932(0.865,1.004) | 0.938(0.870,1.011) |
|  | MET-min/wk ≥1,000 | 16,139 | 606 | 8.68 | 0.845(0.776,0.921) | 0.851(0.781,0.928) |
| MET-min/wk <1,000 | Non-exerciser | 21,304 | 899 | 9.91 | 0.916(0.852,0.984) | 0.919(0.855,0.988) |
|  | MET-min/wk <1,000 | 16,824 | 350 | 4.83 | 0.678(0.607,0.757) | 0.688(0.616,0.769) |
|  | MET-min/wk ≥1,000 | 11,128 | 266 | 5.54 | 0.726(0.641,0.823) | 0.739(0.652,0.838) |
| MET-min/wk ≥1,000 | Non-exerciser | 15,802 | 694 | 10.44 | 0.909(0.838,0.985) | 0.916(0.845,0.993) |
|  | MET-min/wk <1,000 | 9,472 | 263 | 6.42 | 0.754(0.665,0.855) | 0.765(0.675,0.868) |
|  | MET-min/wk ≥1,000 | 16,236 | 391 | 5.71 | 0.68(0.612,0.755) | 0.695(0.625,0.772) |
| **Hip Fracture** | |  |  |  |  |  |
| Non-exerciser | Non-exerciser | 74,647 | 1,201 | 3.77 | 1(Ref.) | 1(Ref.) |
|  | MET-min/wk <1,000 | 20,682 | 225 | 2.48 | 0.919(0.797,1.060) | 0.947(0.821,1.093) |
|  | MET-min/wk ≥1,000 | 16,139 | 165 | 2.33 | 0.834(0.708,0.983) | 0.850(0.722,1.001) |
| MET-min/wk <1,000 | Non-exerciser | 21,304 | 227 | 2.45 | 0.815(0.707,0.940) | 0.832(0.721,0.959) |
|  | MET-min/wk <1,000 | 16,824 | 83 | 1.13 | 0.649(0.519,0.813) | 0.690(0.552,0.864) |
|  | MET-min/wk ≥1,000 | 11,128 | 63 | 1.30 | 0.677(0.525,0.874) | 0.716(0.555,0.925) |
| MET-min/wk ≥1,000 | Non-exerciser | 15,802 | 198 | 2.92 | 0.889(0.764,1.034) | 0.898(0.772,1.045) |
|  | MET-min/wk <1,000 | 9,472 | 70 | 1.69 | 0.758(0.595,0.966) | 0.791(0.621,1.009) |
|  | MET-min/wk ≥1,000 | 16,236 | 124 | 1.79 | 0.799(0.663,0.964) | 0.850(0.704,1.025) |
| *Abbreviation: MET, metabolic equivalents of tasks | | | | | | |
